# Functional and Structural Analysis of a β-Glucosidase Involved in β-1,2-Glucan Metabolism in *Listeria innocua*

**DOI:** 10.1371/journal.pone.0148870

**Published:** 2016-02-17

**Authors:** Masahiro Nakajima, Ryuta Yoshida, Akimasa Miyanaga, Koichi Abe, Yuta Takahashi, Naohisa Sugimoto, Hiroyuki Toyoizumi, Hiroyuki Nakai, Motomitsu Kitaoka, Hayao Taguchi

**Affiliations:** 1 Department of Applied Biological Science, Faculty of Science and Technology, Tokyo University of Science, Noda, Chiba, Japan; 2 Department of Chemistry, Tokyo Institute of Technology, Meguro-ku, Tokyo, Japan; 3 Graduate School of Science & Technology, Niigata University, Nishi-ku, Niigata, Japan; 4 National Food Research Institute, National Agriculture and Food Research Organization, Tsukuba, Ibaraki, Japan; Weizmann Institute of Science, ISRAEL

## Abstract

Despite the presence of β-1,2-glucan in nature, few β-1,2-glucan degrading enzymes have been reported to date. Recently, the Lin1839 protein from *Listeria innocua* was identified as a 1,2-β-oligoglucan phosphorylase. Since the adjacent *lin1840* gene in the gene cluster encodes a putative glycoside hydrolase family 3 β-glucosidase, we hypothesized that Lin1840 is also involved in β-1,2-glucan dissimilation. Here we report the functional and structural analysis of Lin1840. A recombinant Lin1840 protein (Lin1840r) showed the highest hydrolytic activity toward sophorose (Glc-β-1,2-Glc) among β-1,2-glucooligosaccharides, suggesting that Lin1840 is a β-glucosidase involved in sophorose degradation. The enzyme also rapidly hydrolyzed laminaribiose (β-1,3), but not cellobiose (β-1,4) or gentiobiose (β-1,6) among β-linked gluco-disaccharides. We determined the crystal structures of Lin1840r in complexes with sophorose and laminaribiose as productive binding forms. In these structures, Arg572 forms many hydrogen bonds with sophorose and laminaribiose at subsite +1, which seems to be a key factor for substrate selectivity. The opposite side of subsite +1 from Arg572 is connected to a large empty space appearing to be subsite +2 for the binding of sophorotriose (Glc-β-1,2-Glc-β-1,2-Glc) in spite of the higher *K*_m_ value for sophorotriose than that for sophorose. The conformations of sophorose and laminaribiose are almost the same on the Arg572 side but differ on the subsite +2 side that provides no interaction with a substrate. Therefore, Lin1840r is unable to distinguish between sophorose and laminaribiose as substrates. These results provide the first mechanistic insights into β-1,2-glucooligosaccharide recognition by β-glucosidase.

## Introduction

β-1,2-Glucan is a homo-polymer composed of glucose, as are cellulose (β-1,4-glucan) and laminarin (β-1,3-glucan), and is found mainly in some Gram-negative bacteria, such as *Agrobacterium*, *Rhizobium*, *Shinorhizobium*, and *Brucella*, as a cyclic form [1–5]. Cyclic β-1,2-glucan is known as an infectious or commensal factor in animals or plants and is used as a modulator of intracellular osmotic pressure after secretion into the extracellular space and accumulation in the periplasm [4–7]. Linear β-1,2-glucan is also found as an extracellular or periplasmic glucan with degrees of polymerization (DP) of 5–13 possessing β-1,6-glucosyl branches in *Escherichia coli* and *Pseudomonas syringae* [8–10]. In addition, sophorosides are found in some plants [11]. In contrast to the existence of β-1,2-glucan in nature, only a few β-1,2-glucan degrading enzymes has been reported. Though β-1,2-glucan-degrading glucanases and glucosidases induced by β-1,2-glucan have been reported in *Cytophaga* (*Chitinophaga*) *arvensicola*, a Gram negative bacterium, and *Acremonium* sp. 15, a filamentous anamorphic fungus [12,13], their amino acid sequences have not been elucidated.

Recently, the Lin1839 protein from *Listeria innocua* was identified as a 1,2-β-oligoglucan phosphorylase (OGP), an enzyme specific to β-1,2-glucan. The cytosolic Lin1839 enzyme catalyzes reversible phosphorolysis of β-1,2-glucans with DP of 3 or more to produce α-glucose 1-phosphate (G1P) [14], but the enzyme does not act on sophorose (Glc-β-1,2-Glc, Sop_2_). Thus, it is likely that other enzymes are also required for complete dissimilation of β-1,2-glucans. In the gene cluster containing the *lin1839* gene, the *lin1840* gene encodes a putative glycoside hydrolase family (GH) 3 β-glucosidase (BGL) (GenBank accession number: CAC97071.1) [15]. These facts led us to hypothesize that the Lin1840 protein is suitable for Sop_2_ degradation, although there is no experimental evidence supporting this hypothesis.

GH3 is one of the major families containing BGLs along with GH1. BGLs form a large subgroup widely distributed in animals, plants, and microorganisms in GH3 containing *N*-acetyl-β-d-glucosaminidases, α-l-arabinofuranosidases and β-d-xylopyranosidases as well [16]. The substrate recognition residues and their structural positions for non-reducing end glucosides are highly conserved among GH3 BGLs. On the other hand, the substrate recognition sites in other moieties of substrates exhibit great diversification in the family, which leads to a variety of substrate and chain length specificities [17].

Lin1840 forms a clade with closely related homologs phylogenetically [18]. Only two enzymes have been characterized and/or are structurally available in the clade. Metagenomic GH3 β-glucosidase from compost (JMB19063), which is the only structurally available enzyme, was reported to act on cellooligosaccharides (β-1,4-glucooligosaccharides, Cel_n_s) [18], and *Flavobacterium meningosepticum* GH3 β-glucosidase (*Fm*BGL) has been identified as an aryl β-glucosidase [19]. The amino acid sequence identities of these two enzymes with Lin1840 are 42% and 40%, respectively. Arg587, which is one of the main residues comprising subsite +1 in JMB19063, is highly conserved among the closely related homologs (S1 Fig). The presence of a conserved arginine residue important for substrate recognition led us to expect a similar function among Lin1840, JMB19063, and *Fm*BGL. Cel_n_s were the only natural compounds tested as substrates for previous characterization of JMB19063 and *Fm*BGL. However, both enzymes have very low activity on Cel_n_s, whereas they show high activity toward *p*-nitrophenyl-β-d-glucopyranoside (pNP-β-Glc), an artificial substrate [18,19]. This means that previous reports of *Fm*BGL and JMB19063 characterization lack information supporting or denying the hypothesis that Lin1840 is involved in β-1,2-glucan metabolism in *L*. *innocua*. In this study, we describe the characteristics and structure-function relationship of the Lin1840 to provide the first mechanistic insight into recognition of β-1,2-glucooligosaccharides (Sop_n_s) by BGLs.

## Materials and Methods

### Preparation of recombinant Lin1840 and mutants

Gene cloning and overexpression of *lin1840* from *L*. *innocua* Clip11262 and purification of recombinant Lin1840 (Lin1840r) was described in our previous paper [20]. Briefly, the protein fused with a C-terminal His_6_-tag was purified from the cell extract of the transformant using a HisTrap FF crude column (5 ml; GE Healthcare, Buckinghamshire, England), and then was buffered with 50 mM 3-(*N*-morpholino)propanesulfonic acid (Mops) buffer (pH 7.0) using Amicon Ultra 30,000 molecular weight cut-off (Millipore, Billerica, MA, USA) for enzyme assay. The protein was further purified using Superdex 200 (Hiload 16/60; GE Healthcare) for crystallization. The molecular weight of Lin1840r in solution was estimated from the retention time. Ovalbumin (44 kDa), conalbumin (75 kDa), aldolase (158 kDa), ferritin (440 kDa), and thyroglobulin (669 kDa; GE Healthcare) were used as standard proteins. Blue dextran 2000 (2000 kDa; GE Healthcare) was used to determine the void volume of the column. Protein concentration was determined by UV absorbance at 280 nm (extinct coefficient [21] and theoretical molecular weight of Lin1840r are 67770 cm^−1^M^−1^ and 80532.2 Da, respectively). Construction of plasmids for expression of the D270A, E473A, and R572K mutants was performed based on the protocol for a KOD-Plus-Mutagenesis Kit (Toyobo, Osaka, Japan) using pET-28a inserted with *lin1840* as a template, KOD Plus (TOYOBO, Osaka, Japan), and the primers described below. The primer pairs used for amplification of the D270A, E473A, and R572K mutant genes were 5’-TGGGGCGCTGTTGCCGAAGTAATTAATCAC-3’ and 5’-CGCAGAAATAAGTACACCGTCAAACTCCA-3’, 5’-CCCGCCCCATTCATTTTTTTCACCTAGCGC-3’ and 5’-GCGGCAGGAAGTCTTGCTACTATTCG-3’, and 5’-GAGCGCCACAAACACCGGAAAATAAAGG-3’ and 5’-CAGTGCGTAAATGATTATAATAAACTGG-3’ (mutated nucleotides are underlined), respectively. Production and purification of the mutant enzymes were performed in the same way as for the WT.

### Enzyme assays using *p*-nitrophenyl-sugars

The substrate specificity of Lin1840r toward pNP-β-Glc, pNP-β-d-xylopyranoside, pNP-β-d-fucopyranoside, pNP-β-d-arabinofuranoside, and pNP-β-d-galactopyranoside (Sigma Aldrich, St. Louis, MO, USA) was tested. Glycerol was used for stabilization of Lin1840r during the reaction. The reaction mixture comprising 50 mM 2-(*N*-morpholino)ethanesulfonic acid (Mes) buffer (pH 6.0), 5 mM substrate, 15% glycerol and Lin1840r in a total volume (20 μl) was incubated at 20°C for 10 min. After incubation, 10 μl of the reaction mixture was taken and mixed with 90 μl of 0.2 M Na_2_CO_3_ in a 96-well plate (EIA/RIA, plate, 96-well half area; Corning, NY, USA) to stop the reaction. The hydrolytic activity toward pNP-sugars was determined by measuring the absorbance at 405 nm derived from free pNP using a microplate reader (SpectraMax190; Molecular Devices, CA, USA). Various concentrations of pNP (0.005–0.5 mM) were used as standards.

### Kinetic analysis using pNP-β-Glc and β-linked gluco-oligosaccharides

To determine the kinetic parameters of pNP-β-Glc hydrolysis, 70 μl of a reaction mixture comprising 0.5–30 mM pNP-β-Glc, 15% glycerol and 0.42 μg of the enzyme in 50 mM Mes buffer (pH 6.0) was incubated at 20°C for 10 min. Ten μl aliquots of the reaction mixture were taken and then was mixed with 90 μl of 0.2 M Na_2_CO_3_ every 2 min to stop the reaction. Activity was determined as described above. The kinetic parameters were determined by non-linear regression of the data using an equation expressing substrate inhibition: *v*/[E_0_] = *k*_cat_[S]/(*K*_m_+[S]+[S]^2^/*K*_is_) (equation 1), where *v* is the initial velocity of pNP release, [E_0_] the enzyme concentration, and *K*_is_ the substrate inhibition constant. The kinetic analysis regarding β-linked gluco-oligosaccharides was performed using Sop_n_s [14], β-1,2-glucans (average DP 25) [22], Lam_n_s (BIOCON (JAPAN) LTD, Aichi, Japan), Cel_2_ (Wako Pure Chemical Industries, Osaka, Japan), and Gen_2_ (Tokyo Chemical Industry, Tokyo, Japan) as substrates. The reaction mixtures (70 μl) comprising various concentrations of substrates, the enzyme, and 15% glycerol in 50 mM Mes buffer (pH 6.0) were incubated at 20°C for 10 min. At intervals of two minutes, 10 μl aliquots of the mixtures were taken to stop the reaction by heat treatment at 99°C. Then the samples mixed with 90 μl of GOPOD FORMAT KIT (Megazyme, Wicklow, Ireland) were incubated at 45°C for 20 min. When the substrates were Sop_n_ and Lam_n_, 6 μl aliquots of the samples and 114 μl of GOPOD FORMAT KIT were mixed. The amounts of Glc released from oligosaccharides were calculated from absorbance at the 510 nm using 100 μl aliquots of the solutions. The concentrations of Glc released from disaccharides were taken to be half since two Glc molecules were released in hydrolysis of one substrate molecule. The kinetic parameters were determined by fitting to equation 1 or the normal Michaelis-Menten equation.

### Temperature and pH profiles

The reaction conditions and the method for measuring released pNP were the same as described in the kinetic analysis section. The substrate used was 5 mM pNP-β-Glc. The optimum temperature and pH were determined by measuring the activity at various temperatures (0–50°C) and in various pH ranges in the following buffers: sodium acetate (pH 4.0–5.5), Mes (pH 5.5–6.5), Mops (pH 6.5–7.5), 4-(2-hydroxyethyl)piperazine-1-(2-hydroxypropanesulfonic acid) (pH 7.5–8.5), and *N*-cyclohexyl-2-aminoethanesulfonic acid (pH 8.5–9.0). The thermal and pH stabilities were determined from the residual hydrolytic activity at 20°C after incubation of Lin1840r (0.1 mg/ml) at various temperatures in 50 mM Mes buffer (pH 6.0) for 1 h, and in 50 mM various buffers with the pH ranges described above at 20°C for 1 h, respectively. The incubated enzyme solutions were diluted at least 20 times with the reaction solution.

### Inhibition kinetics

Gluconic acid δ-lactone (GDL) (Wako Pure Chemical Industries), isofagomine d-tartrate (IFG) (Toronto Research Chemicals Inc., Toronto, Canada), and 1-deoxynojirimycin (DNJ) (Wako Pure Chemical Industries) were used as inhibitors. The inhibitory effect on hydrolytic activity toward pNP-β-Glc was measured with various substrates (1.5–10 mM) and inhibitor concentrations in 50 mM Mes buffer (pH 6.0) and 15% glycerol at 20°C. Kinetic parameters were determined using Grafit 7.0.3 by non-linear regression of the data using equations of competitive inhibition: *v*/[E_0_] = *k*_cat_ [*S*]/{*K*_m_ (1 + [I]/*K*_i_) + [*S*]} or mixed-type inhibition: *v*/[E_0_] = *k*_cat_ [S]/{*K*_m_ (1 + [I]/*K*_i_) + [S] (1 + [I]/*K*_i_’)}, where *v* is the initial velocity of pNP release, [E_0_] the enzyme concentration, *K*_i_ the competitive inhibition constant, and *K*_i_’ the noncompetitive inhibition constant.

### Crystallography

Crystallization of the WT and D270A mutant was performed by the hanging-drop vapor-diffusion method. As described previously [20], 1 μl of 10 mg/ml Lin1840r in 5 mM Mops (pH 7.0) mixed with 1 μl of reservoir solution comprising 15% (v/v) glycerol, 0.17 M Li_2_SO_4_, 0.085 M Tris-HCl (pH 9.0), and 25.5% (w/v) PEG4000 was incubated at 25°C for 1 week for crystallization. Crystals were soaked in the reservoir solution supplemented with each ligand for 1 h to obtain complex structures. Crystals of WT were soaked in 150 mM Glc, 5 mM GDL, or 5 mM IFG. D270A crystals were soaked in 100 mM Sop_2_, 100 mM Sop_3_, 50 mM Lam_2_, 300 mM Cel_2_, or 300 mM Gen_2_. The crystals were cooled and then kept at 100 K in a nitrogen-gas stream during data collection. A set of X-ray diffraction data for the crystal was collected using a CCD detector (ADSC Quantum 210r) on a beamline AR-NW12A at Photon Factory (Tsukuba, Japan). The diffraction data set was processed using iMosflm [23]. A model structure of Lin1840r was predicted using SWISS-MODEL (http://swissmodel.expasy.org/) [24] based on the A chain of *Hv*ExoI (PDB code; 1EX1), and then used as a search model for molecular replacement. Molecular replacement was performed using MOLREP [25] to determine initial phases. Automated model building was performed using ARP/wARP [26]. Manual model building and refinement were performed using Coot [27] and Refmac5 [28], respectively. Quality check of the structures was performed using wwPDB validation server (http://wwpdb-validation.wwpdb.org/validservice/). The figures were prepared using PyMOL (DeLano Scientific; http://www.pymol.org). The buried surface area was calculated with the protein-protein interaction interface server (PISA; http://www.ebi.ac.uk/msd-srv/prot_int/pistart.html) [29].

## Results

### General properties of Lin1840r

The amino acid sequence of Lin1840 includes no predicted N-terminal signal peptide, as judged from SignalP 4.0 analysis (http://www.cbs.dtu.dk/services/SignalP/) [30], suggesting that the enzyme is localized in the cytosol. On size-exclusion chromatography, a recombinant Lin1840 protein was eluted as a 150 kDa one, suggesting that it is dimeric. The enzyme did not show significantly decreased catalytic activity on incubation up to 20°C in the pH range of 5.0–9.0, but drastically lost the activity at 40°C. The enzyme exhibited maximal catalytic activity at 37°C and pH 6.0. Asp270 and Glu473 in Lin1840r are predicted to be a catalytic nucleophile and a catalytic acid/base, respectively, according to primary sequence alignment with *Fm*BGL [31,32].

### Substrate specificity

The substrate specificity of Lin1840r as to glycone was determined using several *p*-nitrophenyl (pNP)-β-d-monosaccharides. The enzyme showed no activity toward any of those examined (less than 0.01 U/mg) except for pNP-β-Glc, indicating that it specifically acts upon β-glucosides. The kinetic parameters of the enzyme toward pNP-β-Glc were *k*_cat_ = 48 ± 3 (s^−1^), *K*_m_ = 3.1 ± 0.3 (mM), and *k*_cat_/*K*_m_ = 15 ± 1 (mM^−1^ s^−1^). To investigate the linkage position specificity, kinetic parameters for β-linked gluco-disaccharides were determined (Table 1). Lin1840r showed large *k*_cat_ values for Sop_2_ and Lam_2_, while the *k*_cat_ values for Cel_2_ and gentiobiose (Glc-β-1,6-Glc, Gen_2_) were less than 1% of those for Sop_2_ and laminaribiose (Glc-β-1,3-Glc, Lam_2_). The *K*_m_ values for Sop_2_ and Lam_2_ were similarly small, while the *K*_m_ values for Cel_2_ and Gen_2_ were approximately 7 and 13 times higher than that for Sop_2_, respectively. Consequently, the enzyme exhibited comparable *k*_cat_/*K*_m_ values for Sop_2_ and Lam_2_, while the values for Cel_2_ and Gen_2_ were both less than 0.05% of that for Sop_2_. The kinetic parameters for Sop_2_ and Lam_2_ were similar to those for pNP-β-Glc. Then kinetic parameters for Sop_n_s were determined. The *k*_cat_ value for Sop_3_ was similar to that for Sop_2_, but the *k*_cat_ values for Sop_4_ and Sop_5_ were less than one-tenth of that for Sop_2_. The *K*_m_ values for Sop_3-5_ were 5 or more times higher than that for Sop_2_. Consequently, the catalytic efficiency decreased with the increase in DP remarkably. In the case of β-1,3-glucooligosaccharides (Lam_n_s), in contrast, the *k*_cat_, *K*_m_ or *k*_cat_/*K*_m_ value did not markedly change with increasing DP, unlike in the case of Sop_n_s. The enzyme did not show significant hydrolytic activity toward β-1,2-glucan (reaction velocity, less than 0.01 U/mg in the presence of 1 mM substrate).

**Table 1:** Kinetic parameters of Lin1840r for β-linked gluco-oligosaccharides and pNP-β-Glc.

Substrate^a^			*k*_cat_ (s^−1^)	*K*_m_ (mM)	*K*_is_ (mM)	*k*_cat_/*K*_m_ (s^−1^mM^−1^)
WT	Sop_2_	(β-1,2)^b^	41 ± 4	2.0 ± 0.3	14 ± 4	21 ± 1
	Sop_3_		56 ± 11	15 ± 4		3.8 ± 0.3
	Sop_4_		3.5 ± 0.9	10 ± 4		0.34 ± 0.04
	Sop_5_		1.0 ± 0.4	11 ± 6		0.098 ± 0.020
	Lam_2_	(β-1,3)^b^	23 ± 1	2.7 ± 0.2		8.8 ± 0.4
	Lam_3_		11 ± 1	3.7 ± 0.3		2.9 ± 0.1
	Lam_4_		14 ± 5	5.4 ± 2.5	2.5 ± 1.3	2.6 ± 0.2
	Lam_5_		12 ± 5	5.5 ± 2.8	2.7 ± 1.6	2.1 ± 0.2
	Cel_2_	(β-1,4)^b^	0.24 ± 0.01	27 ± 1		0.0090 ± 0.0003
	Gen_2_	(β-1,6)^b^	0.076 ± 0.007	14 ± 2	150 ± 50	0.0054 ± 0.0005
	pNP-β-Glc		48 ± 3	3.1 ± 0.3		15 ± 1
R572K	Sop_2_		15 ± 1	21 ± 2		0.69 ± 0.03
	Lam_2_		14 ± 5	9.2 ± 0.6		1.5 ± 0.1
	Cel_2_		0.094 ± 0.005	68 ± 8		0.0014 ± 0.0005
	Gen_2_		0.22 ± 0.01	48 ± 5		0.0045 ± 0.0003

^*a*^ Substrate concentrations used, 0.5–8 mM (Sop_2_), 0.5–8 mM (Sop_3_), 1–8 mM (Sop_4_), 1–8 mM (Sop_5_), 0.5–8 mM (Lam_2_), 0.5–8 mM (Lam_3_), 0.5–6 mM (Lam_4_), 0.75–6 mM (Lam_5_), 1–70 mM (Cel_2_), 1–80 mM (Gen_2_), and 0.5–30 mM (pNP-β-Glc) for WT and 1–15 mM (Sop_2_), 0.5–10 mM (Lam_2_), 5–100 mM (Cel_2_), and 1–90 mM (Gen_2_) for R572K.

^*b*^ Linkages are shown in parentheses.

### Inhibition kinetics

The inhibition modes and constants for six inhibitors as to the hydrolytic activity toward *p*NP-β-Glc are summarized in Table 2. Three glucosidase inhibitors, GDL, IFG, and DNJ, consistently exhibited competitive-type inhibition with *K*_i_ values of less than 1 mM. IFG showed the strongest inhibition among all the inhibitors examined, the *K*_i_ value being 4.1 μM. While the *K*_i_ value of Lin1840r for IFG was similar to that of *Aspergillus aculeatus* BGL1 (*Aa*BGL1) (14 μM), the *K*_i_ value of Lin1840r for DNJ (290 μM) was over 100 times higher than that of *Aa*BGL1 (2.4 μM) [33]. On the other hand, Glc, Cel_2_, and Gen_2_ consistently showed mixed-type inhibition (Table 2). The *K*_i_ and *K*_i_’ values for Cel_2_ were more than 5 times higher than those for Glc and Gen_2_.

**Table 2:** Inhibition constants for inhibitors.

Inhibitor	Mode	*K*_i_ (mM)	*K*_i_’ (mM)
GDL^a^	Competitive	0.30 ± 0.04	
IFG^b^	Competitive	0.0041 ± 0.0005	
DNJ^c^	Competitive	0.29 ± 0.02	
Glc^d^	Mixed	3.8 ± 0.6	90 ± 42
Cel_2_^e^	Mixed	21 ± 5	470 ± 270
Gen_2_^f^	Mixed	2.8 ± 0.4	38 ± 9

^*a*^Activity toward pNP-β-Glc (1.5–10 mM) in the presence of 0, 0.5, 1, and 5 mM GDL was used.

^*b*^Activity toward pNP-β-Glc (1.5–10 mM) in the presence of 0, 0.005, 0.025, and 0.05 mM IFG was used.

^*c*^Activity toward pNP-β-Glc (1.5–10 mM) in the presence of 0, 0.5, 1, and 2 mM DNJ was used.

^*d*^Activity toward pNP-β-Glc (2–10 mM) in the presence of 0, 5, 10, and 20 mM Glc was used.

^*e*^Activity toward pNP-β-Glc (2–10 mM) in the presence of 0, 10, 30, and 50 mM Cel_2_ was used.

^*f*^Activity toward pNP-β-Glc (2–10 mM) in the presence of 0, 5, 10, and 20 mM Gen_2_ was used.

### Overall structure

**Fig 1:**
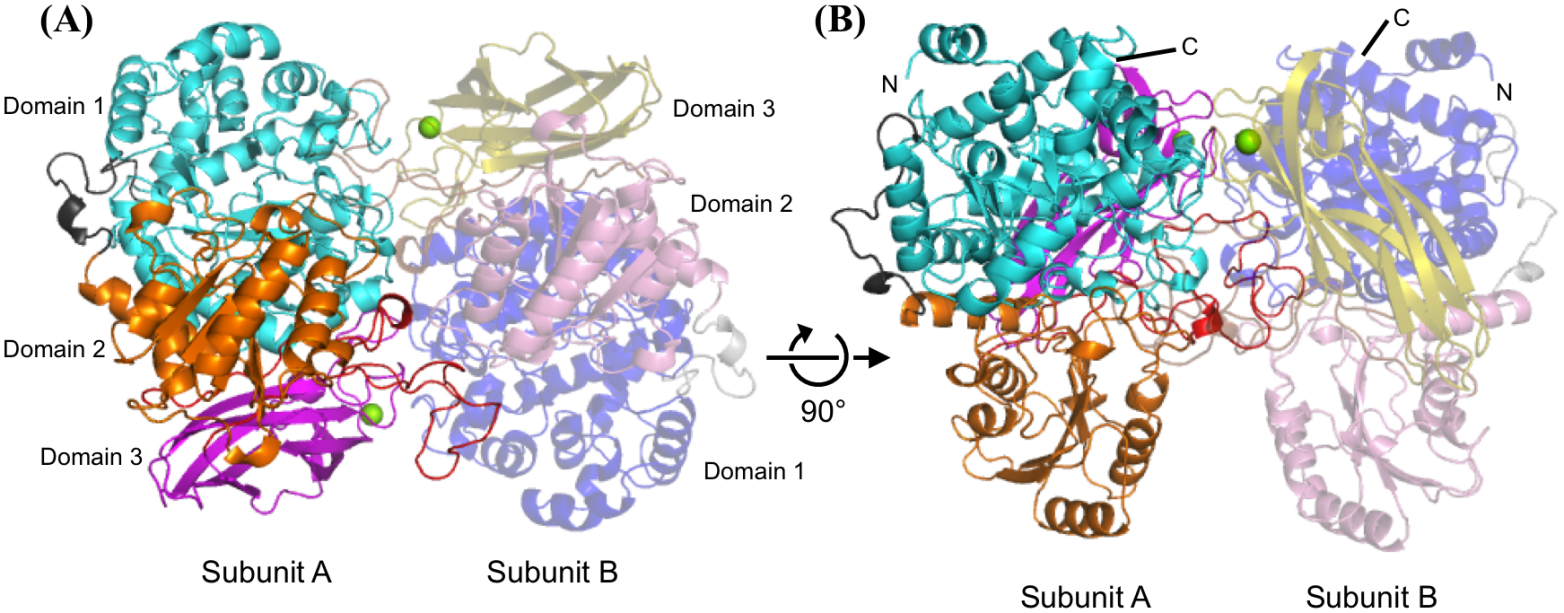
Overall structure of Lin1840r. **(A)** Lin1840r is a dimer composed of subunits A and B. Domains 1 ((β/α)_8_ triose phosphate isomerase barrel fold, amino acids 1–339), 2 ((α/β)_6_ sandwich fold, amino acids 360–543), and 3 (fibronectin type III (FnIII) family, amino acids 605–723) in subunit A are presented in *cyan*, *orange*, and *magenta*, respectively. The loop extending to the active center in subunit B (amino acids 543–604), and the linker region that connects domains 1 and 2 (amino acids 340–359) are shown in *red* and *black*, respectively. The corresponding three domains, loop, and linker in subunit B are presented in semitransparent *blue*, *pink*, and *yellow*, *brown*, and *gray*, respectively. Mg^2+^ ions are shown as *green* spheres. N-terminal and C-terminal regions of both subunits are denoted by N and C, respectively.

The crystal structure of apo wild-type (WT) Lin1840r was determined at 1.8 Å resolution (S1 Table). The structure showed that Lin1840r forms a dimer of identical subunits each composed of three domains (Fig 1A). The structure of Lin1840r was compared with those of two GH3 enzymes, JMB19063 and barley GH3 β-glucan-exohydrolase (*Hv*ExoI), exhibiting the highest similarity based on the Z-score with the Dali server (http://ekhidna.biocenter.helsinki.fi/dali_server/) [34] (S2 Table). Lin1840r shows a similar overall structure to that of JMB19063. A loop (amino acids 568–598) in Lin1840r extends into the active site of the other subunit to form a part of the substrate pocket as in the case of the corresponding loop of JMB19063 (Fig 1 and S2A Fig). Therefore, the formation of the dimer is predicted to be important for the catalysis. Contrarily, *Hv*ExoI is a monomeric enzyme whose catalytic site is composed of a single subunit [35]. Lin1840r is composed of three domains based on Pfam (http://pfam.xfam.org/) [36]. There are some differences in each domain from those of JMB19063. In domain 1, the N-terminal region of Lin1840r (amino acids 34–53) forms an α helix, whereas it forms a loop in JMB19063 (amino acids 29–48) [18] (S2B Fig). In domain 2, the helix and loop (amino acids 435–455) extending to the active site in JMB19063 are missing in Lin1840r (S2B Fig). In domain 3, clear electron density was observed (S2C Fig). The metal ion obviously undergoes six-coordination with the side chain of Asp648, the backbone carbonyl of Thr650, and four water molecules. The metal ion is suggested to be Mg^2+^ according to the server for checking metal ion binding (http://csgid.org/csgid/metal_sites/, [37]) (S2 Table), although a Mg^2+^ ion was not added to Lin1840r in any step of sample preparation.

### Subsite −1 of Lin1840r

The active center of Lin1840r is located at the interface of domains 1 and 2 as in the cases of known GH3 BGLs. The two predicted catalytic residues, Asp270 and Glu473, occupy similar positions to the corresponding Asp residues (catalytic nucleophile) and Glu residues (catalytic acid/base), respectively, of the known enzymes (S3 Fig). In fact, the D270A and E473A mutants showed no detectable hydrolytic activity toward pNP-β-Glc. This result supports the assignment of Asp270 and Glu473 as the catalytic nucleophile and catalytic acid/base residues, respectively. A distance between side chain carboxyl oxygen atoms of Asp270 and Glu473 is approximately 6.0 Å, suggesting that the enzyme follows retaining mechanism.

The Lin1840r-Glc complex structure was determined to understand the substrate recognition at subsite −1. Six residues (Asp91, Arg149, Lys191, His192, Asp270, and Glu473) constitute subsite −1 and form hydrogen bonds with the Glc molecule (S3 Fig). These residues can be well superimposed on the corresponding residues of the known structures of GH3 BGLs.

### Complexes with inhibitors

**Fig 2:**
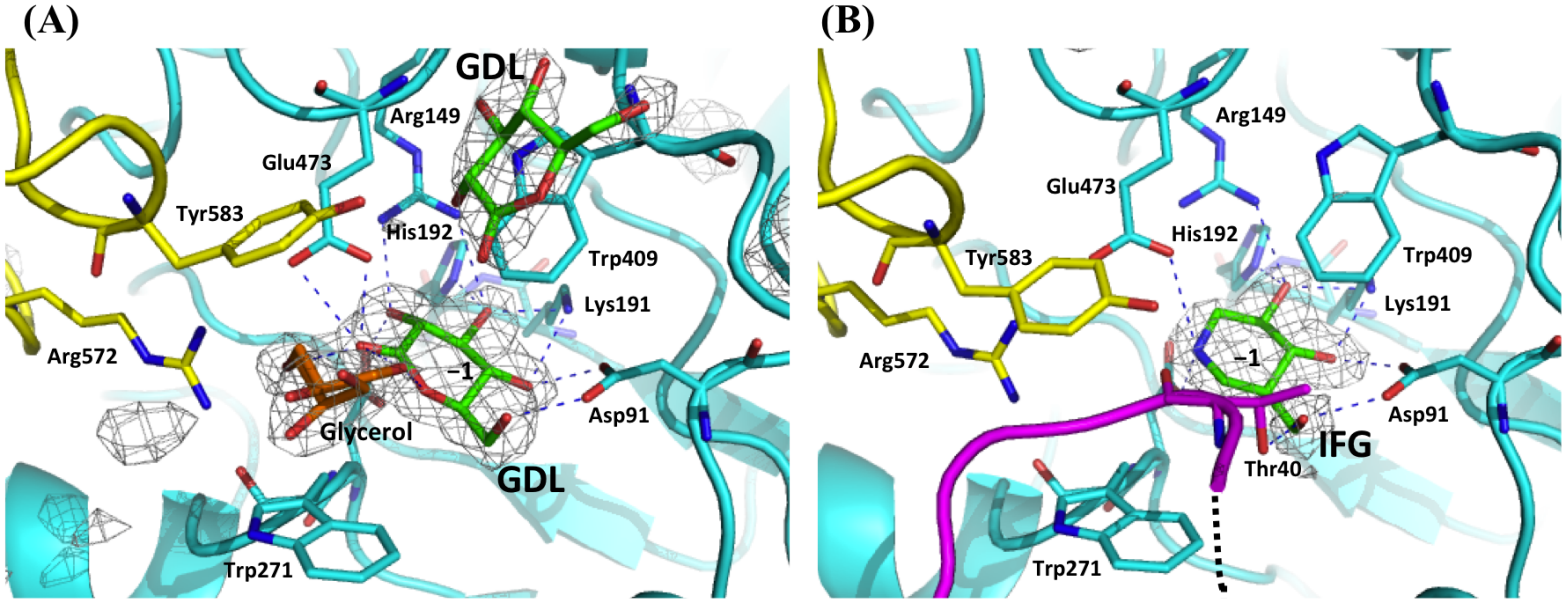
Complexes with GDL (A), and IFG (B). The two subunits comprising the catalytic pocket are shown in *cyan* and *yellow*. Residues involved in ligand binding are shown as sticks. The Leu39–Met47 loop seen in the IFG complex is shown in *magenta* and the disordered loop region (Gly32–Glu38) are shown in a *black dotted* line. Inhibitors and glycerol are represented as *green* and *orange* sticks, respectively. *F*_o_−*F*_c_ electron density maps of GDL and IFG are shown as a *gray* mesh (contoured at 3.0σ and 4.0σ, respectively). GDL and glycerol (A) and IFG (B) were omitted for calculation of the *F*_o_−*F*_c_ maps. Hydrogen bonds are presented as *blue dotted* lines.

To clarify the binding modes of inhibitors, Lin1840r-inhibitor complex structures were determined. In complex structures with IFG and GDL, which are Glc analogs, the ligands are located at the same position as Glc (Fig 2A, 2B and S3 Fig), suggesting that IFG and GDL compete with substrates for subsite −1. In the GDL complex, electron densities of glycerol molecules were observed between the aromatic rings of Tyr583 and Trp271 (Fig 2A). A GDL molecule can be fitted to a large electron density near Trp409 (outside the active center) and undergo hydrophobic stacking with Trp409, but the orientation of the molecule is obscure (Fig 2A). The structure of the GDL complex is not substantially different from those of the apo WT and Glc complex. In the case of the IFG complex structure, on the other hand, an unwound helix (amino acids 36–47) in domain 1 is inserted between the aromatic rings of Tyr583 and Trp271. Thr40 in this loop forms a hydrogen bond with the 6-OH group of IFG (Fig 2B). This loop might be involved in substrate binding in solution.

### Complex structures with Sop_2_ and Lam_2_

**Fig 3:**
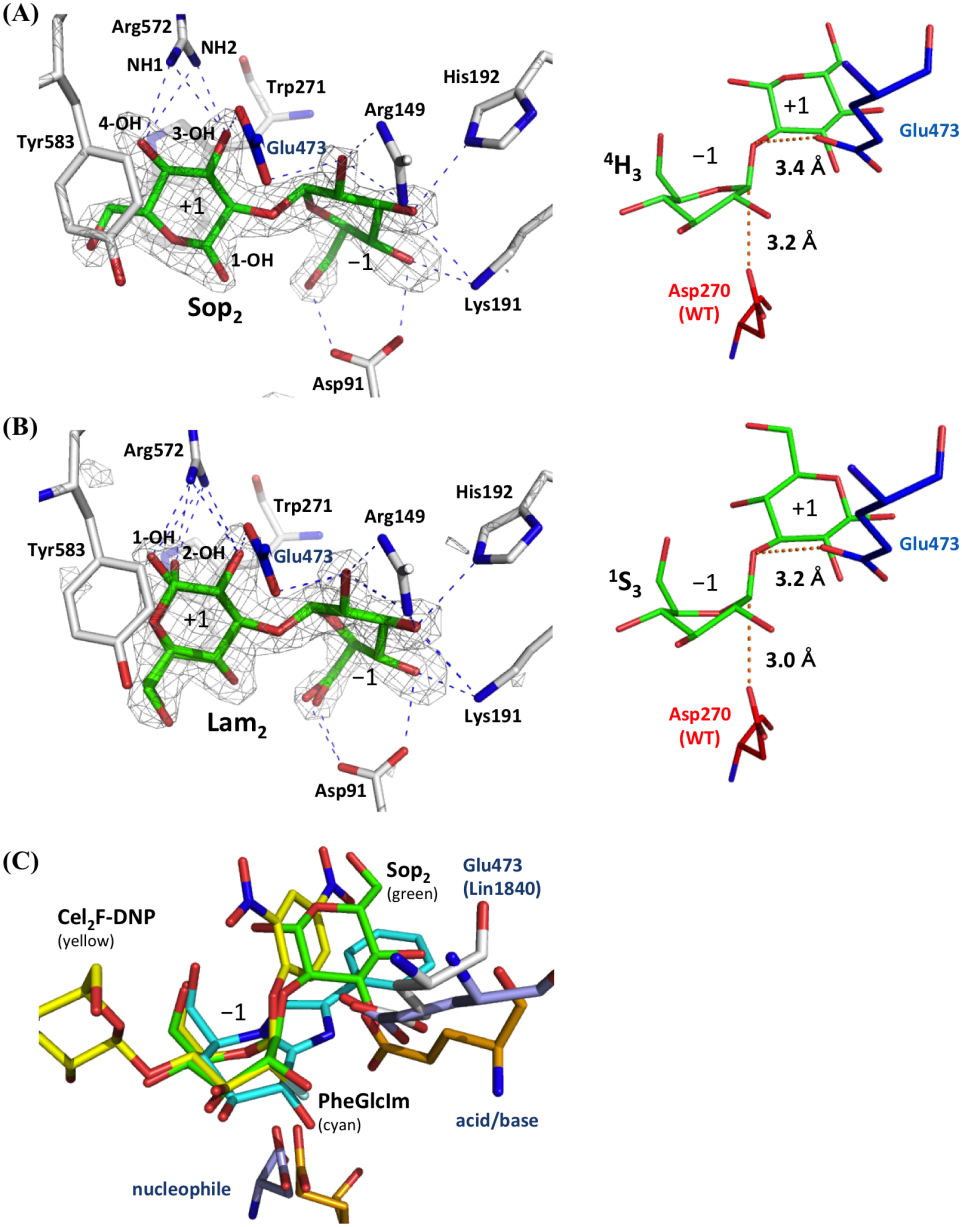
D270A-Sop_2_ and D270A-Lam_2_ complexes. **(A, B)** Substrate pockets of D270A-Sop_2_ (A) and D270A-Lam_2_ (B) complexes. Ligands are shown as *green* sticks. Acid-base residues are shown in *blue*. (*Left*) Electron density maps of ligands. The *F*_o_−*F*_c_ electron density maps of the ligands are presented as a *gray* mesh (contoured at 3.0σ). Sop_2_ and Lam_2_ are omitted for calculation of the *F*_o_−*F*_c_ maps. Several hydroxy groups of ligands are labeled. Hydrogen bonds are presented as *blue dotted* lines. Lam_2_ in the D270A-Lam_2_ complex **(B)** is a mixture of both anomers. Distances between Arg572 and ligands are summarized in S3 Table. (*Right*) Conformations of ligands at subsite −1. The position of the catalytic nucleophile (Asp270) determined on superpositioning with apo WT is shown in *red*. The distances between two atoms linked by *red dotted* lines are given. **(C)** Comparison of conformation of Sop_2_ with those of ligands in Michaelis complex and transition state-like complexes at subsite −1. Sop_2_, Cel_2_F-DNP in *Ba*Cel5A (Michaelis complex), and PheGlcIm in *Hv*ExoI (transition state) are shown in *green*, *yellow*, and *cyan* sticks, respectively. Catalytic residues of Lin1840r, *Hv*ExoI, and *Ba*Cel5A are shown in *white*, *bright orange*, and *light blue*, respectively.

In order to understand the recognition mechanism of Sop_2_ and Lam_2_, the complex structures with these disaccharides were determined using the nucleophile D270A mutant of Lin1840r. The electron density of Sop_2_ was clearly observed in the D270A structure on soaking in 100 mM Sop_2_ and fitted only with the β-anomer (Fig 3A left). The significant electron density of a ligand was also observed on soaking in 50 mM Lam_2_ and clearly fitted with both anomers of Lam_2_ (Fig 3B left). The Glc moieties of both Sop_2_ and Lam_2_ at subsite −1 are located at almost the same positions as the ligands in WT-inhibitors complexes despite the replacement of the catalytic nucleophile with alanine. The reducing end Glc moieties of Sop_2_ and Lam_2_ are consistently stacked on Trp271 and Tyr583, and form four hydrogen bonds with Arg572 (S3 Table), suggesting that these three residues constitute subsite +1. The fact that Tyr583 and Arg572 are derived from a loop in the adjacent subunit indicates the participation of both subunits in formation of the catalytic pocket.

The results of Cremer-Pople ring pucker parameters calculator analysis (http://www.ric.hi-ho.ne.jp/asfushi/) [38] showed that the pyranose rings of Sop_2_ and Lam_2_ at subsite −1 are ^4^H_3_ and ^1^S_3_, respectively. Since proposed itinerary of ring conformation to the substrate-enzyme intermediate in *Hv*ExoI is ^4^C_1_ (pre-Michaelis complex) ⬄ ^1^S_3_ (Michaelis complex) ⬄ ^4^E (^4^H_3_) (transition state) ⬄ ^4^C_1_ (intermediate) [39,40], conformation of Lam_2_ is considered to correspond with Michaelis complex. Sop_2_ complex is also considered as Michaelis complex in spite of its conformation, since Sop_2_ is not superimposed with glucophenylimidazole (PheGlcIm) known as a transition-like analog in *Hv*ExoI [40] but with ligands known as Michaelis complex, such as 2',4'-dinitrophenyl-2-deoxy-2-fluro-β-d-cellobioside (Cel_2_F-DNP) in GH5 *Bacillus agaradhaerens endo-*β-1,4-glucanase (*Ba*Cel5A), a retaining enzyme [41] (Fig 3C). In addition, the Sop_2_ molecule does not possess a trigonal anomeric center necessary to be judged as transition state. In the Sop_2_ and Lam_2_ complexes, the angles defined by the oxygen atom of the glycosidic bond, the anomeric carbon atoms in the ligands, and the carboxyl group in the catalytic nucleophile are 165° and 164°, respectively, as in the case of GlcNAc-MurNAc in a nucleophile mutant of GH3 *B*. *subtillus N*-acetyl-β-d-glucosaminidase and Cel_2_F-DNP in *Ba*Cel5A [41,42]. Dihedral angles of Sop_2_ and Lam_2_ (O2-C1-O5-C5 in non-reducing end) are 93.4° and 88.3°, respectively, implying that the lone pairs on the both endocyclic oxygen atoms almost face antiperiplanar to the scissile bonds. These facts suggest that the nucleophile is able to mount an in-line attack on the anomeric carbon (Fig 3A and 3B right) [43]. The distances between the catalytic nucleophile and the anomeric carbon at subsite −1 are 3.2 Å and 3.0 Å, respectively, and the distances between the catalytic acid-base and oxygen atoms of the glycosidic bond are 3.4 Å and 3.2 Å, respectively (Fig 3A and 3B right). These distances are within the possible range of the reaction [44]. These facts suggest that the ligands are positioned, as they would be in catalytically active complexes. This is the first report of a Michaelis complex for GH3 BGLs, though pre-Michaelis complexes have been reported for *Hv*ExoI [39,40].

The anomeric hydroxy group of reducing end glucosides in Sop_2_ is exposed to the solvent, in the vicinity of Asp91. In the case of Lam_2_, the hydroxy group is exposed outside the substrate pocket, this being consistent with similar activity among Lam_n_s.

### Complexes with Sop_3_, Cel_2_, and Gen_2_

Soaking of the D270A mutant crystal in 100 mM Sop_3_ resulted in clear observation of electron density corresponding to the middle Glc moiety of Sop_3_ at subsite +1 (S4A Fig). The electron densities of glycoside moieties at both the non-reducing and reducing ends might be derived from Sop_3_ but they were so weak that interpretation was difficult. The electron density of the Glc moiety was not observed at subsite −1. In the Cel_2_ and Gen_2_ complex structures, electron densities of Glc moieties were also both observed at subsite +1 (S4B and S4C Fig). These Glc moieties were thought to be the non-reducing ends of Cel_2_ and Gen_2_ due to the orientation of the hydroxy group participating in the glycosidic linkage (S4B and S4C Fig), suggesting that Cel_2_ or Gen_2_ binds to the enzyme non-productively. In the both complexes, Glc molecules were observed only in molecule A. This might be due to impurity in quite high concentrations of soaked substrates. In the Cel_2_ complex, Trp409 stacks on another Glc moiety unlike in the cases of the Sop_3_ and Gen_2_ complexes (S4 Fig).

### Kinetic analysis of the R572K mutant

It is suggested that Arg572 is an important residue for the recognition of Sop_2_, since it forms multiple hydrogen bonds with substrates at subsite +1 (Fig 3A and 3B left). We therefore characterized the R572K mutant. The kinetic parameters of the R572K mutant enzyme as to β-linked gluco-disaccharides are summarized in Table 1. The *K*_m_ value of the mutant enzyme for Sop_2_ was 10 times higher than that of the WT enzyme. The mutant enzyme also showed increased *K*_m_ values for the other substrates, and 1.5–3.0 times smaller *k*_cat_ values for Sop_2_, Lam_2_, and Cel_2_. As a result, the *k*_cat_/*K*_m_ value of the R572K mutant for Sop_2_ was over 25 times smaller, while the extent of reduction of the *k*_cat_/*K*_m_ value for Lam_2_ caused by the mutation was approximately 6 times. This indicates that Arg572 is important for substrate binding, especially for Sop_2_.

### Comparison of substrate pockets

**Fig 4:**
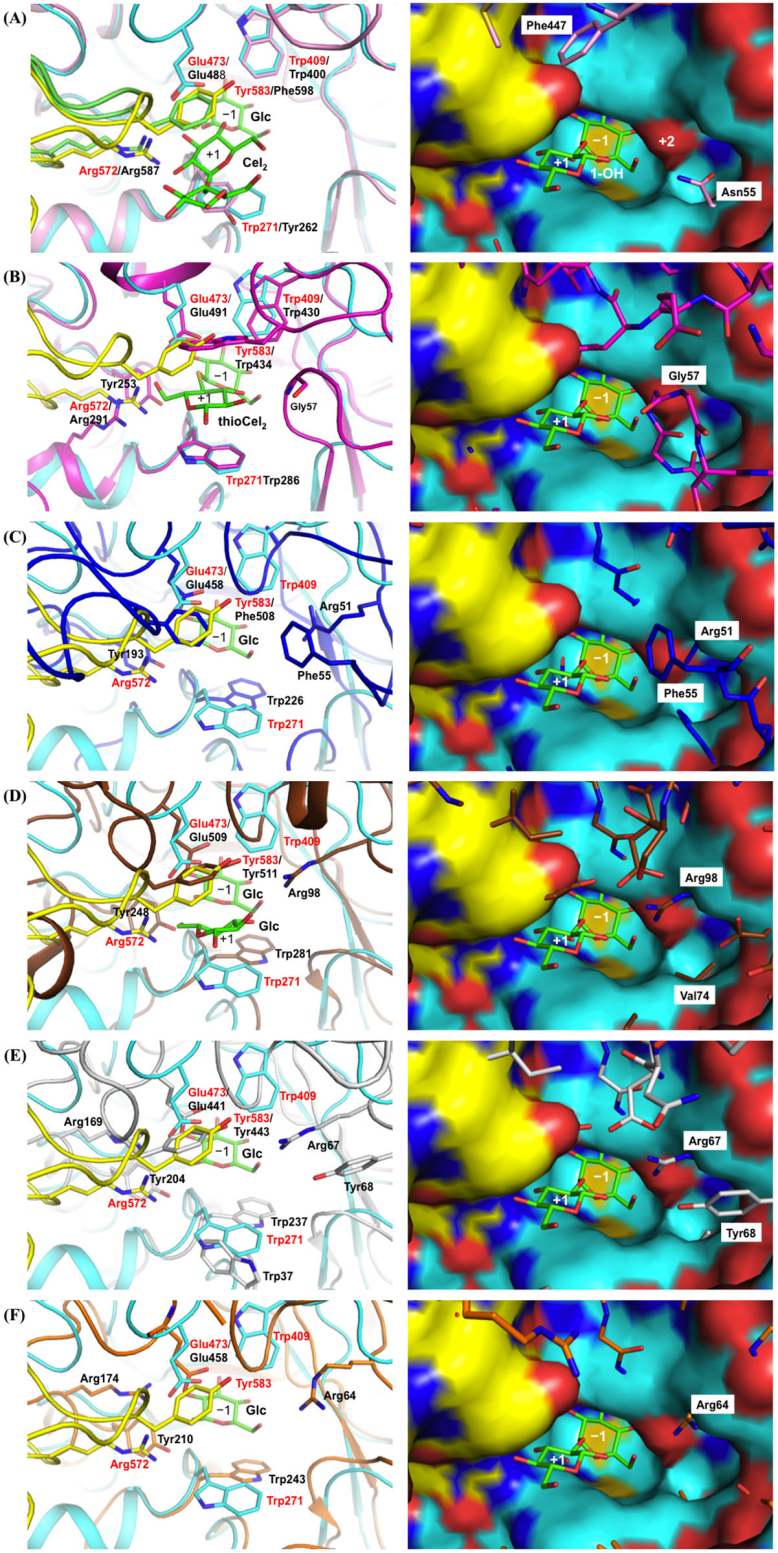
Comparison of the active centers in Lin1840r and GH3 BGLs. Apo WT Lin1840r is superimposed on **(A)** JMB19063, **(B)** *Hv*ExoI (PDB code 1IEX), **(C)** *Km*BglI (PDB code 3AC0), **(D)** *Aa*BGL1 (PDB code 4IIG), **(E)** *Tr*Cel3A (PDB code 3ZYZ), and **(F)** *Tn*Bgl3B (PDB code 2X41). (*Left*) Domains 1 and 2 of apo WT Lin1840r are shown in *cyan*, and the loop extending from the other subunit is shown in *yellow*. The corresponding domains and loop of JMB19063 are shown in *pink* and *green*, respectively. *Hv*ExoI, *Km*BglI, *Aa*BGL1, *Tr*Cel3A, and *Tn*Bgl3B are colored *magenta*, *blue*, *brown*, *gray*, and *orange*, respectively. Residues constituting the subsites are represented as sticks. The residue names in Lin1840r and the other enzymes are shown in *red* and *black*, respectively. (*Right*) The D270A-Sop_2_ complex was used in place of the apo WT enzyme. D270A is shown as a surface representation. The superimposed GH3 BGLs are shown as sticks. Sop_2_ in the D270A-Sop_2_ complex is represented as a *green* stick. All ligands shown in the *left* figures are omitted in the *right* figures. **(A)** An anomeric hydroxy group of Sop_2_ is labeled.

In order to investigate the importance of Arg572 and subsite +2, the substrate pocket of Lin1840r was compared with those of JMB19063, a homolog in the same clade as Lin1840, and homologs in the different clade from Lin1840. Trp271, Tyr583, and Arg572 constitute subsite +1 in Lin1840r. They can be well superimposed on the corresponding residues in JMB19063 (Tyr262, Phe598, and Arg587, respectively) (Fig 4A). Try583 and Arg572 are derived from the other subunit, as observed for the corresponding residues of JMB19063. Contrarily, in the other structurally known GH3 BGLs, each subsite +1 comprises one subunit.

The Trp271 residue is conserved in other structurally available GH3 BGLs, including *Hv*ExoI, β-glucosidase from *Kluyveromyces marxianus* (*Km*BglI) [45], *Aa*BGL1 [33], β-glucosidase from *Trichoderma reesei* (*Tr*Cel3A) [46] which act on a variety of substrates containing cellobiose unlike Lin1840r, and aryl β-glucosidase from *Thermotoga neapolitana* (*Tn*Bgl3B) [47]. The corresponding residues in most GH3 BGLs are aromatic amino acids. The Trp286 residue in *Hv*ExoI stacks on the Glc moiety of the ligand at subsite +1, while Trp226 of *Km*BglI, Trp281 of *Aa*BGL1, Trp237 of *Tr*Cel3A, and Trp243 of *Tn*Bgl3B participate in recognition of ligands at subsite −1 (Fig 4B–4F).

At the position corresponding to the Tyr583 residue, most structurally known GH3 BGLs have an aromatic amino acid, while the *Tn*Bgl3B structure lacks an aromatic residue probably due to disorder of the corresponding region. The Tyr583 residue and the corresponding residues of JMB19063, *Km*BglI, *Aa*BGL1, and *Tr*Cel3A (Phe598, Phe508, Tyr511, and Trp443, respectively) are consistently located on loops extending from the acid-base catalyst residue side (Fig 4A and 4C–4E). On the other hand, Trp434 in *Hv*ExoI is on the loop extending from the opposite side (Fig 4B). Lin1840r and JMB19063 consistently lack this loop, and instead have empty spaces sufficiently large for glycoside binding (Fig 4A). This space is considered to be subsite +2, since the anomeric hydroxy group of reducing end glucosides in Sop_2_ faces the space (Fig 4A *right*). The spaces are filled with loops in *Km*BglI, *Aa*BGL1, and *Tr*Cel3A (Fig 4C–4E).

The Arg572 residue in the substrate pocket is located at a similar position to Arg291 of *Hv*ExoI (Fig 4B). However, Arg291 does not correspond with the Arg572 residue in terms of primary sequence, and *Hv*ExoI does not have any corresponding loop for the Arg572 residue. The Arg291 residue is localized farther away from the center of the catalytic pocket than Arg572 (Fig 4B). *Km*BglI, *Aa*BGL1, *Tr*Cel3A, and *Tn*Bgl3B possess no residue that corresponds to Arg572 or Arg291 (Fig 4C–4F). Gly57 in the loop of *Hv*ExoI (amino acids 54–66) participates in substrate recognition on the opposite side of subsite +1 from Arg291 [48] (Fig 4B). *Km*BglI, *Aa*BGL1, and *Tr*Cel3A possess Phe55, Arg98, and Arg67, respectively, at the position corresponding to Gly57 (Fig 4C–4E). On the other hand, Lin1840r has no corresponding loop (Fig 4A).

## Discussion

In this study, the hydrolytic activity of Lin1840r toward Sop_n_s was determined since the *lin1839* gene in the same gene cluster as *lin1840* encodes an enzyme specific to β-1,2-glucan. Lin1840r showed obvious preference for Sop_2_ among Sop_n_s (Table 1). Considering that Lin1839 phosphorolyzes β-1,2-glucan with DP 3 or more to produce G1P but does not act on Sop_2_ [14], Lin1840 and Lin1839 cooperatively metabolize β-1,2-glucan in the cytosol. Since phosphorylation of glucose without the use of ATP is beneficial for energy acquisition, it is advantageous that BGLs show strong preference for substrates on which phosphorylases do not act. These facts suggest that Lin1840 is a BGL for Sop_2_ degradation.

Nevertheless, structural analysis showed that Lin1840r possesses a sufficiently large space, which appears to be subsite +2, for access of Sop_3_. This space is needed only for binding of Sop_n_s, as judged from the orientations of the anomeric hydroxy groups of Sop_2_ and Lam_2_ in Lin1840r structures (Fig 3), and thioCel_2_ (Fig 4B) [35] and methyl β-thiogentiobiose (PDB ID, 3WLP) in *Hv*ExoI structures. The corresponding spaces are filled in other structurally available GH3 BGLs except JMB19063 (Fig 4A–4E). However, the presence of Sop_3_ accessible space at subsite +2 cannot explain the kinetic result that Lin1840r showed a much higher *K*_m_ value toward Sop_3_ than that toward Sop_2_. The conformation of Sop_3_ is likely related to the difference in the *K*_m_ values. According to molecular dynamics simulation, Sop_3_ adopts a stable conformation that includes an intramolecular hydrogen bond between the 3-OH group of the reducing end glucoside and the oxygen atom of the pyranose ring or 6-OH group of the non-reducing end glucoside [49]. These intramolecular hydrogen bonds in Sop_3_ have to be removed and the glycosidic bond of Sop_3_ at the reducing end also has to be distorted for productive binding of Sop_3_. Overall, subsite +2 might be an evolutionary relic of specialization of Lin1840 from a Sop_n_s degrading enzyme to one specialized at Sop_2_ degradation.

**Fig 5:**
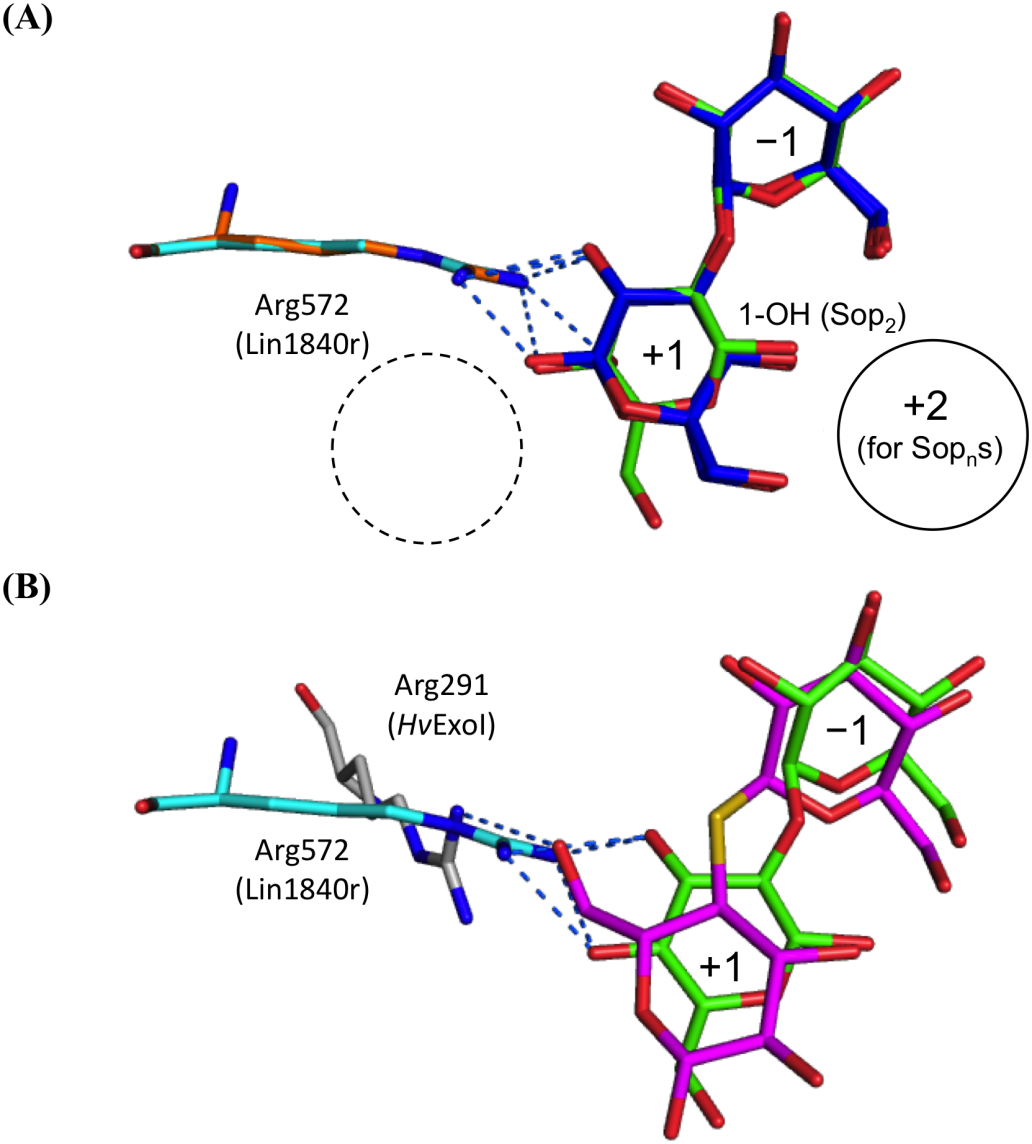
Comparison of conformations of ligands in Lin1840r and *Hv*ExoI. Hydrogen bonds are represented as *blue dotted* lines. Subsites −1, +1, and +2 are labeled. **(A)** Comparison of Sop_2_ and Lam_2_ in the D270A mutant. Arg572 and Sop_2_ in the D270A-Sop_2_ complex are shown in *cyan* and *green*, respectively. Arg572 and Lam_2_ of both anomers in the D270A-Lam_2_ complex are colored *orange* and *blue*, respectively. Putative subsite +2 for Sop_n_s and potential subsite +2 for Lam_n_s are shown in a solid and dotted circle, respectively. An anomeric hydroxy group of a reducing-end glucoside in Sop_2_ is labeled. **(B)** Comparison of Sop_2_ in the D270A mutant and thioCel_2_ in *Hv*ExoI. The residue and ligand in the D270A-Sop_2_ complex are shown in the same as in **(A)**. Arg291 and thioCel_2_ in *Hv*ExoI-thioCel_2_ are presented in *gray* and *magenta*, respectively.

In spite of the estimated function of Lin1840, Lin1840r shows comparable activity toward Lam_2_ with Sop_2_. The presence of Arg572 at subsite +1 and the space of subsite +2 are important features for substrate recognition. Arg572 forms many hydrogen bonds with Sop_2_ or Lam_2_ at subsite +1, and thereby likely compensates for the lack of any hydrogen-bond interaction with the substrates on the subsite +2 side (Figs 3A and 5A). The binding modes of Sop_2_ and Lam_2_ are apparently very similar in the Lin1840r complexes. The structures of the bound Sop_2_ and Lam_2_ molecules only differ in the substituting groups of reducing end glucosides on the subsite +2 side (Fig 5). This observation thus indicates that Lin1840r is not able to distinguish between Sop_2_ and Lam_2_. BGLs from *C*. *arvensicola* and *Acremonium* sp. 15 induced by β-1,2-glucan show similar substrate specificities for β-linked gluco-disaccharides to Lin1840 [12, 13]. They might have similar structural features to Lin1840r, though their amino acid sequences are unavailable.

Unlike Lam_2_, Arg572 effectively excludes Cel_2_ as a substrate. This can be explained by comparison of the D270A-Sop_2_ and *Hv*ExoI-thiocellobiose (thioCel_2_) complex structures. Arg291 in *Hv*ExoI, which corresponds to Arg572 in Lin1840r, is located farther from the center of the substrate pocket than Arg572 (Figs 4B and 5B). The 6-OH group of the reducing end glucoside of thioCel_2_ forms a hydrogen bond with Arg291. However, the distance between Arg572 and the 6-OH of the glucoside in thioCel_2_ is so close as to cause steric hindrance (Fig 5B). Actually, Cel_2_ binds to Lin1840r in the non-productive form. In addition, other structurally available GH3 BGLs (*Tn*Bgl3B, *Tr*Cel3A, *Aa*BGL1 and *Km*BglI), which lack a residue corresponding to Arg572 (Fig 4C–4F), consistently show sufficient hydrolytic activity toward Cel_2_ [45,46,47,50]. These observations suggest that Arg572 accounts for much higher *K*_m_ value for Cel_2_ than those for Sop_2_ and Lam_2_. Considering that Sop_2_ and Lam_2_ complexes mimic Michaelis complex, Arg572 is also related with differences in *k*_cat_ values between Cel_2_ and Sop_2_/Lam_2_. Thus, Arg572 is important for substrate specificity in Lin1840r.

The Arg572 residue is highly conserved among closely related homologs such as JMB19063 and *Fm*BGL. Nevertheless, JMB19063 is thought to be involved in cellulose degradation and *Fm*BGL is described as an aryl β-glucosidase. JMB19063 shares structural features important for substrate specificity with Lin1840. Therefore, we compared the substrate specificities of the three enzymes. The specific activity of JMB19063 toward Cel_2_ (0.1 mM Cel_2_ at 50°C) estimated from the substrate consumption is only 0.077 (U/mg), whereas JMB19063 shows high hydrolytic activity toward pNP-β-Glc (11.8 U/mg on 0.1 mM pNP-β-Glc) [18]. The specific activities of Lin1840r for pNP-β-Glc and Cel_2_ (0.1 mM at 20°C) are 1.12 (U/mg) and 8.9 × 10^−4^ (U/mg), respectively. In addition, *Fm*BGL shows similar levels of kinetic parameters for pNP-β-Glc (*k*_cat_ = 39.1 s^−1^, *K*_m_ = 0.49 mM) to Lin1840r but at least 100 times lower activity toward Cel_2_, as judged from preliminary results, than toward pNP-β-Glc [19,32]. Thus, the three enzymes show a similar tendency in substrate specificity as to pNP-β-Glc and Cel_2_. It should be noted that Lin1840r shows comparable activity toward Sop_2_ and Lam_2_ as glycoside hydrolases, while the activities of JMB19063 and *Fm*BGL toward Sop_2_ and Lam_2_ have not been reported. Moreover, the structure of the Lin1840r-Cel_2_ complex was compared with that of the JMB19063-Cel_5_ complex, which contains Glc in subsite −1 and Cel_2_ outside subsite −1 [18] (Fig 4A). This complex is the same as the Cel_2_ complex of Lin1840r in that subsites +1 and −1 are filled with separate molecules (S4B Fig). Moreover, while ThioCel_2_ in the *Hv*ExoI-thioCel_2_ complex forms the intrinsically stable conformation of Cel_2_, glucoside at subsite +1 in the Cel_5_-soaked JMB19063 complex is inverted compared to that of ThioCel_2_ (Figs 4A and 5A). Thus, the Cel_5_-soaked JMB19063 complex seems to be in the non-productive form, as in the case of the Cel_2_-Lin1840r complex. Overall, it would not be surprising if JMB19063 and *Fm*BGL show similar substrate specificity to Lin1840r.

This study strongly suggests that Lin1840 is a BGL for Sop_2_ degradation, and that Arg572 and subsite +2 are the key factors for its substrate specificity. This is not only a significant revelation in the field of β-1,2-glucan metabolizing enzymes but also evoke the need for reevaluation of the functions of closely related homologs with Lin1840 possessing arginine residues corresponding with the Arg572.

## Supporting Information

S1 Fig Multiple alignment of closely related homologs with Lin1840. Multiple alignment was performed using T-COFFEE multiple alignment server (http://tcoffee.vital-it.ch/apps/tcoffee/index.html) [51]. The amino acid sequences are selected evenly among the clade of Lin1840. The amino acid sequence of JMB19063 is based on the PDB (accession number, 3U48). The GenBank accession number of *Fm*BGL is AAB66561.1. Uncharacterized proteins are presented as accession numbers. AEB30119.1, AEE54288.1, AEW02328.1, EEV33587.1, ELK47769.1, ERI92988.1, and ETT38232.1 are the GenBank accession numbers of homologous genes from *Carnobacterium* sp. 17–4, *Haliscomenobacter hydrossis* DSM 1100, *Niastella koreensis* GR20-10, *Enterococcus gallinarum* EG2, *Halobacillus* sp. BAB-2008, *Clostridiales bacterium* oral taxon 876, and *Paenibacillus* sp. FSL R5-808, respectively. 515950490 and 544876686 are the Geneinfo identifier numbers of homologous genes from *Paenisporosarcina* sp. TG-14 and *Virgibacillus* sp. CM-4, respectively. Conserved residues are indicated by *asterisks*. Arg572 in Lin1840 is indicated by an *asterisk* in inverted monochrome.
(TIF)

S2 Fig Comparison of Lin1840r and JMB19063. Domains 1, 2, and 3 of Lin1840r are presented in *cyan*, *orange*, and *magenta*, respectively. The corresponding domains of JMB19063 (PDB code, 3U48) are in *light brown*, *blue*, and *brown*, respectively. The linker region of Lin1840r (amino acids 340–359) is shown in *black*. The loop region (amino acids 543–604) in Lin1840r and the corresponding loop in JMB19063 are labeled and shown in *red* and *green*, respectively. Mg^2+^ and Ca^2+^, and water are shown as *green*, *red*, and *light blue* spheres, respectively. **(A-B)** Superpositioning of the dimers of both Lin1840r and JMB19063. **(A)** Both subunits B are presented in semitransparent. **(B)** Left monomers rotated by 90° in the direction of the Y-axis. The N-terminal and C-terminal regions of both subunits are denoted by N and C, respectively. **(C)** Metal ion binding sites of Lin1840r. Residues involved in binding of metal ions are labeled and represented as sticks. Residues forming inter-subunit hydrogen bonds are shown as sticks. Hydrogen bonds are depicted as *blue dotted* lines. *F*_o_−*F*_c_ electron density maps of metal ions and coordinating atoms are shown as a *gray* mesh (contoured at 3.0σ).
(TIF)

S3 Fig Superimpositioning of subsites −1 of GH3 BGLs. All residues and ligands are shown as sticks. Lin1840r, JMB19063 (PDB ID, 3U48), *Hv*ExoI (PDB ID, 1IEQ), and *Km*BglI (PDB ID, 3AC0) are colored *cyan*, *green*, *orange*, and *magenta*, respectively. Glc molecules in JMB19063, *Hv*ExoI, and *Km*BglI are shown in *blue*, *pink*, and *olive*, respectively. The amino acid residues in Lin1840r are labeled.
(TIF)

S4 Fig Complex structures of D270A with Sop_3_ (A), Cel_2_ (B), and Gen_2_ (C). Residues constituting subsites are shown as sticks. The *F*_o_−*F*_c_ electron density maps of the ligands are represented as a *gray* mesh (contoured at 3.0σ). Glc molecules were omitted for calculation of the *F*_o_−*F*_c_ maps. The color usage is as in Fig 2. Glc molecules are fitted to electron densities and shown in *green* stick. Glc moieties of the ligands are positioned at subsite +1 (A), subsite −1 and +1, and vicinity of Trp409 (B), and subsite −1 and +1 (C), respectively. (B, C) Regions derived from molecule A and B are shown in *cyan* and *yellow*, respectively.
(TIF)

S1 Table Data collection and refinement statistics. (TIF)

S2 Table Comparison of overall structures of Lin1840r and two other GH3 enzymes. (TIF)

S3 Table Distances between Arg572 and ligands in D270A-Sop_2_ and -Lam_2_ complexes. (TIF)
