# Supplementary figures and images for: Functional and Structural Analysis of a β-Glucosidase Involved in β-1,2-Glucan Metabolism in Listeria innocua
Source: PLoS One. 2016 Feb 17;11(2):e0148870. doi: 10.1371/journal.pone.0148870 (PMC4757417; doi:10.1371/journal.pone.0148870)

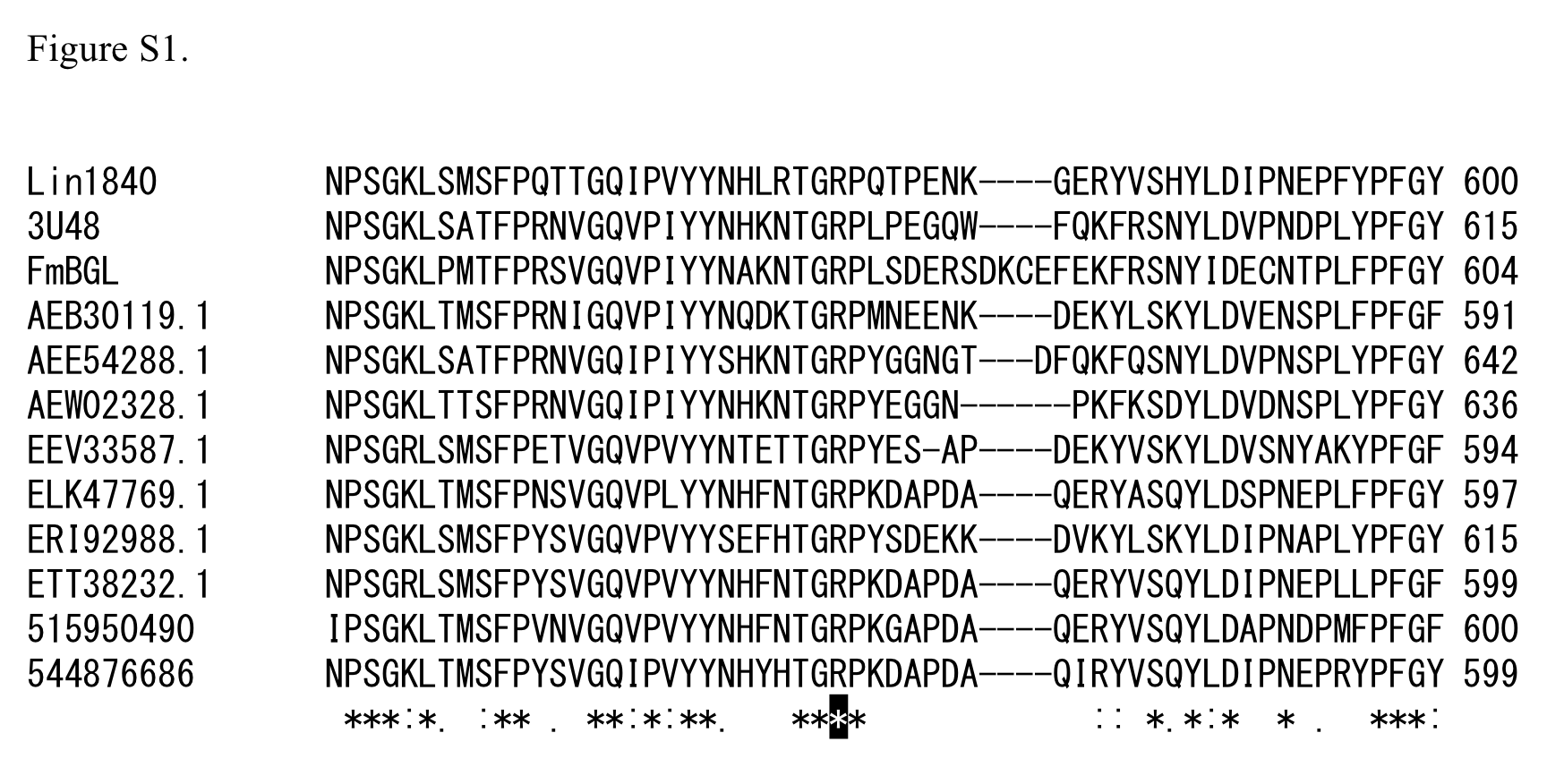

Supplement: S1 Fig — Multiple alignment was performed using T-COFFEE multiple alignment server (http://tcoffee.vital-it.ch/apps/tcoffee/index.html) [51]. The amino acid sequences are selected evenly among the clade of Lin1840. The amino acid sequence of JMB19063 is based on the PDB (accession number, 3U48). The GenBank accession number of FmBGL is AAB66561.1. Uncharacterized proteins are presented as accession numbers. AEB30119.1, AEE54288.1, AEW02328.1, EEV33587.1, ELK47769.1, ERI92988.1, and ETT38232.1 are the GenBank accession numbers of homologous genes from Carnobacterium sp. 17–4, Haliscomenobacter hydrossis DSM 1100, Niastella koreensis GR20-10, Enterococcus gallinarum EG2, Halobacillus sp. BAB-2008, Clostridiales bacterium oral taxon 876, and Paenibacillus sp. FSL R5-808, respectively. 515950490 and 544876686 are the Geneinfo identifier numbers of homologous genes from Paenisporosarcina sp. TG-14 and Virgibacillus sp. CM-4, respectively. Conserved residues are indicated by asterisks. Arg572 in Lin1840 is indicated by an asterisk in inverted monochrome. (TIF) [file pone.0148870.s001.tif]

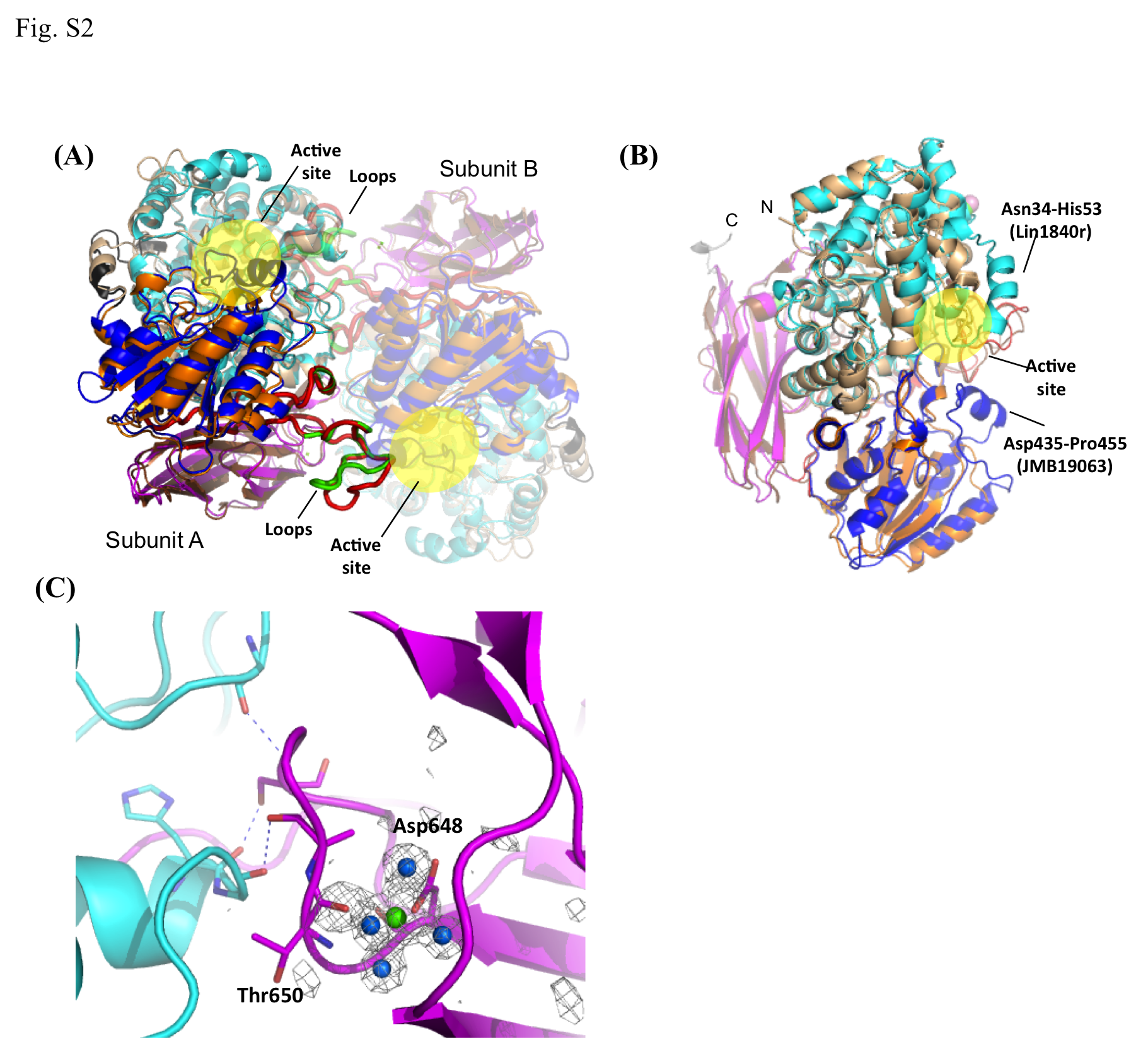

Supplement: S2 Fig — Domains 1, 2, and 3 of Lin1840r are presented in cyan, orange, and magenta, respectively. The corresponding domains of JMB19063 (PDB code, 3U48) are in light brown, blue, and brown, respectively. The linker region of Lin1840r (amino acids 340–359) is shown in black. The loop region (amino acids 543–604) in Lin1840r and the corresponding loop in JMB19063 are labeled and shown in red and green, respectively. Mg2+ and Ca2+, and water are shown as green, red, and light blue spheres, respectively. (A-B) Superpositioning of the dimers of both Lin1840r and JMB19063. (A) Both subunits B are presented in semitransparent. (B) Left monomers rotated by 90° in the direction of the Y-axis. The N-terminal and C-terminal regions of both subunits are denoted by N and C, respectively. (C) Metal ion binding sites of Lin1840r. Residues involved in binding of metal ions are labeled and represented as sticks. Residues forming inter-subunit hydrogen bonds are shown as sticks. Hydrogen bonds are depicted as blue dotted lines. Fo−Fc electron density maps of metal ions and coordinating atoms are shown as a gray mesh (contoured at 3.0σ). (TIF) [file pone.0148870.s002.tif]

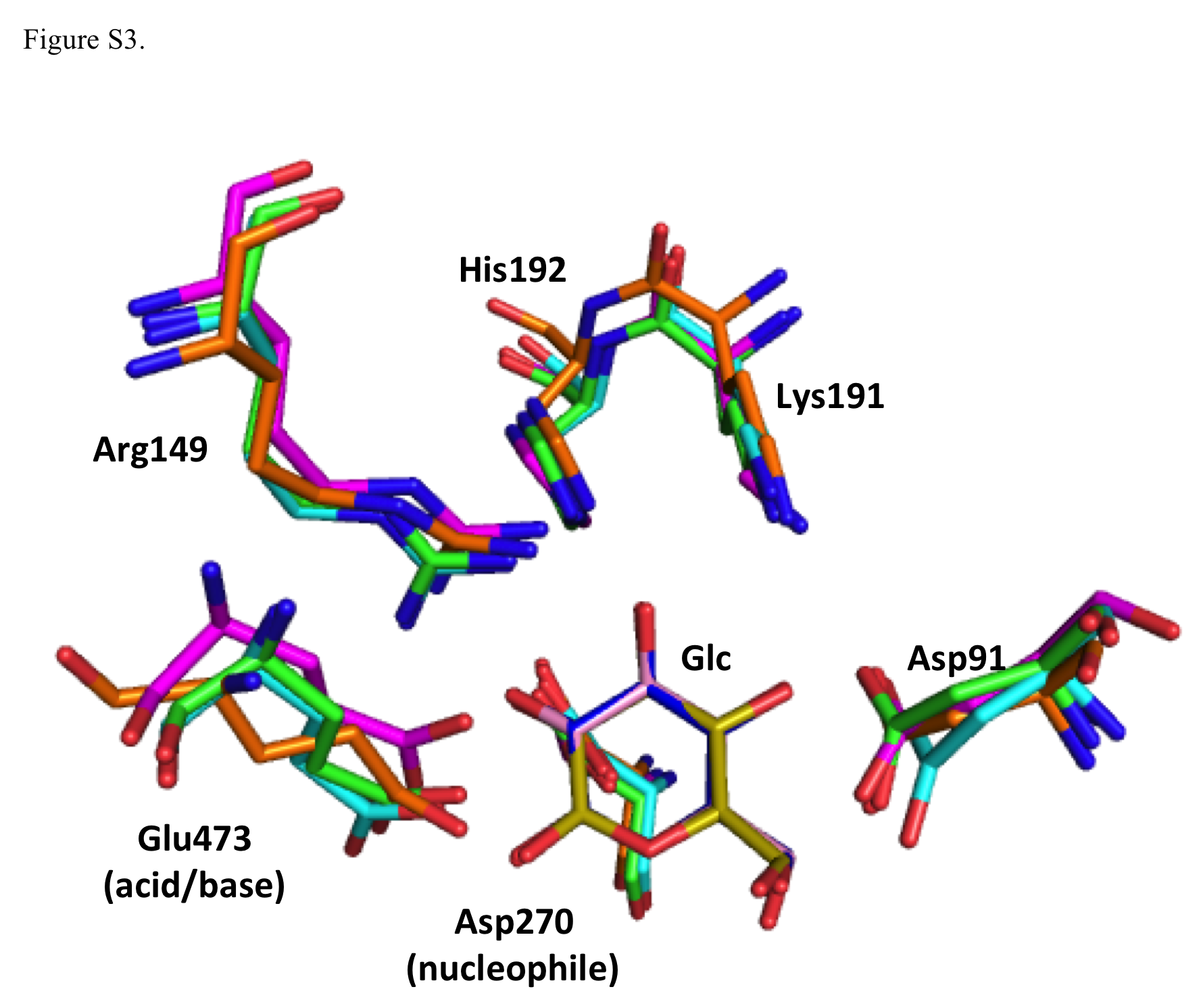

Supplement: S3 Fig — All residues and ligands are shown as sticks. Lin1840r, JMB19063 (PDB ID, 3U48), HvExoI (PDB ID, 1IEQ), and KmBglI (PDB ID, 3AC0) are colored cyan, green, orange, and magenta, respectively. Glc molecules in JMB19063, HvExoI, and KmBglI are shown in blue, pink, and olive, respectively. The amino acid residues in Lin1840r are labeled. (TIF) [file pone.0148870.s003.tif]

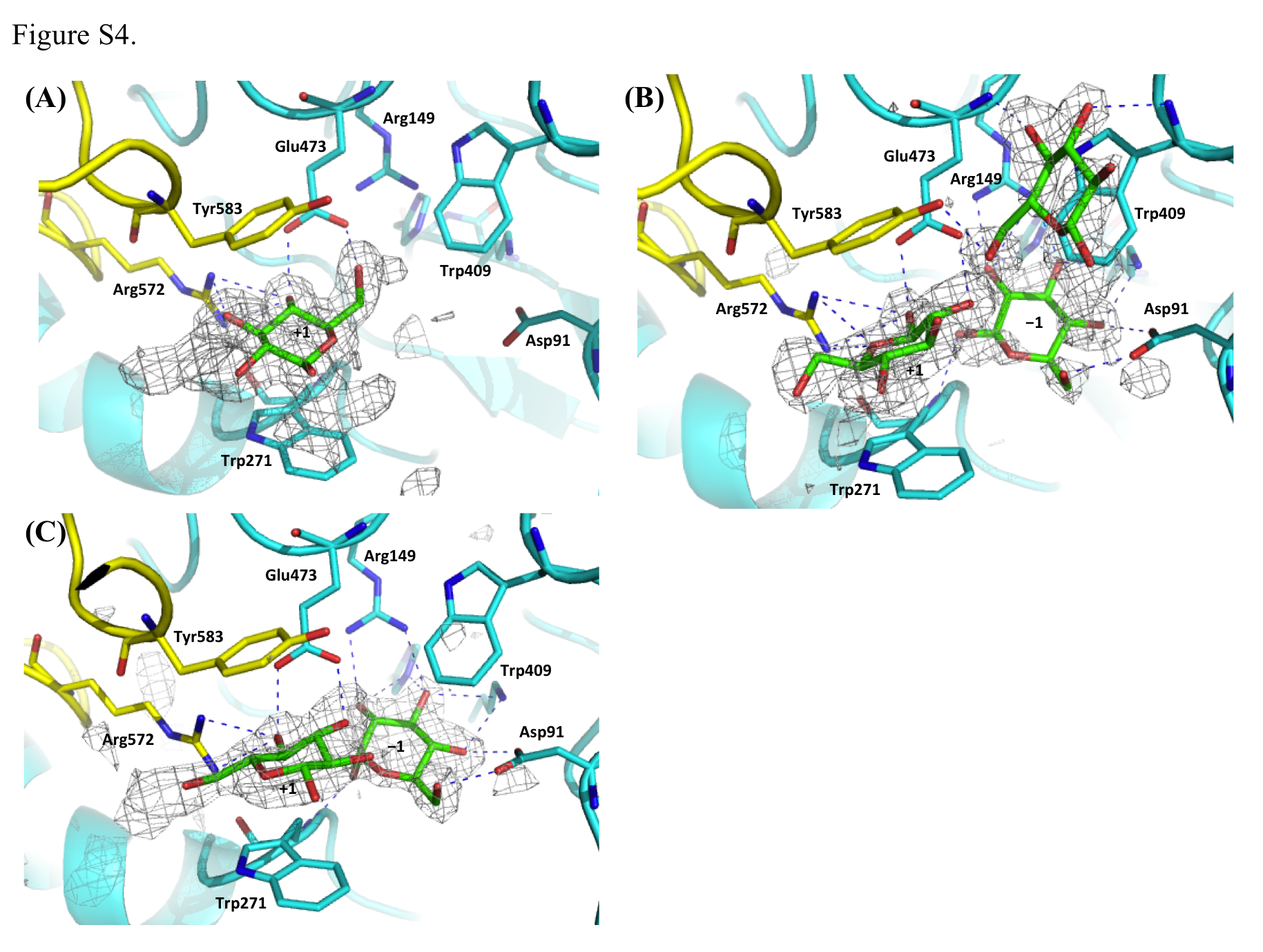

Supplement: S4 Fig — Residues constituting subsites are shown as sticks. The Fo−Fc electron density maps of the ligands are represented as a gray mesh (contoured at 3.0σ). Glc molecules were omitted for calculation of the Fo−Fc maps. The color usage is as in Fig 2. Glc molecules are fitted to electron densities and shown in green stick. Glc moieties of the ligands are positioned at subsite +1 (A), subsite −1 and +1, and vicinity of Trp409 (B), and subsite −1 and +1 (C), respectively. (B, C) Regions derived from molecule A and B are shown in cyan and yellow, respectively. (TIF) [file pone.0148870.s004.tif]

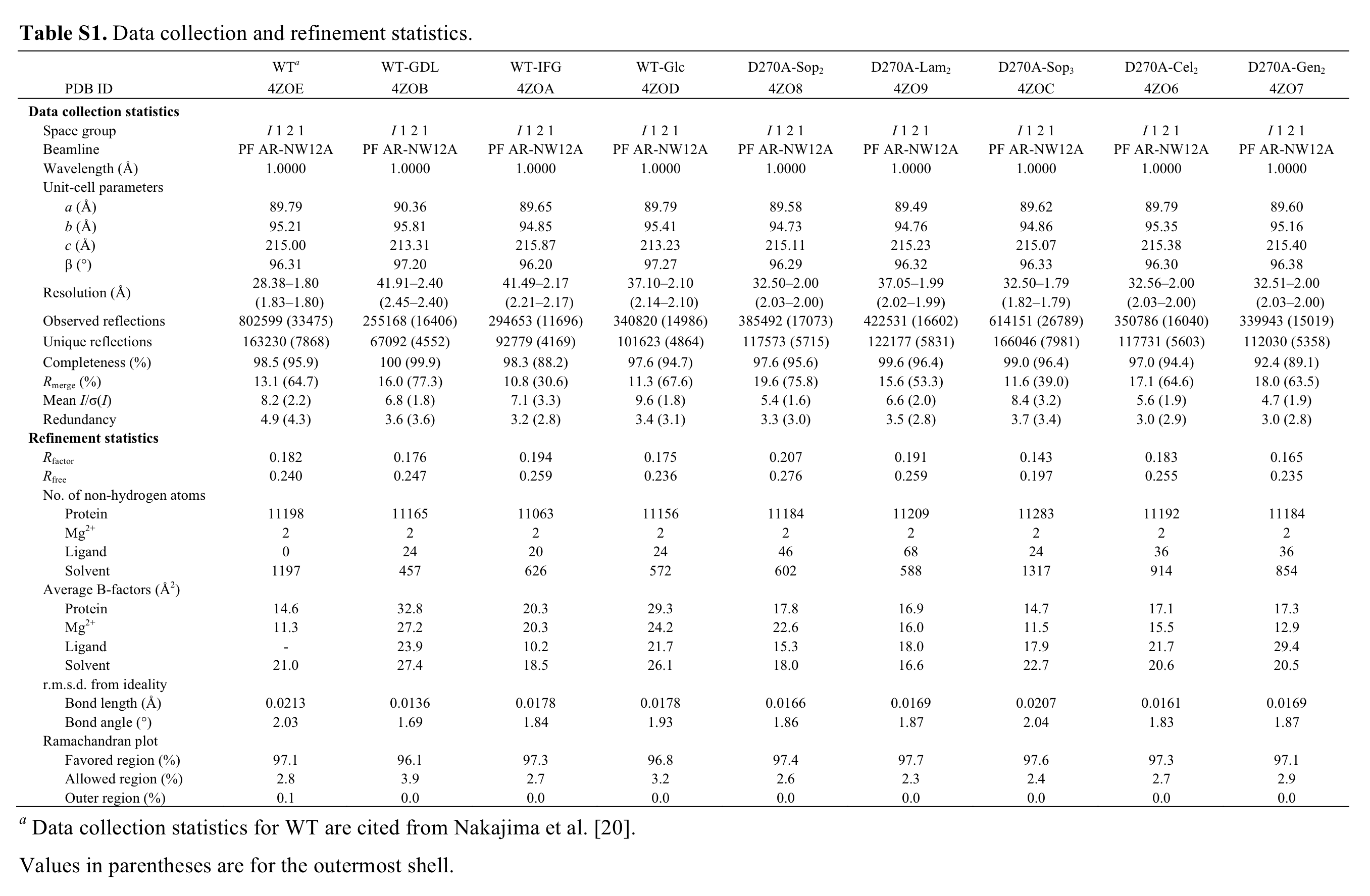

Supplement: S1 Table — (TIF) [file pone.0148870.s005.tif]

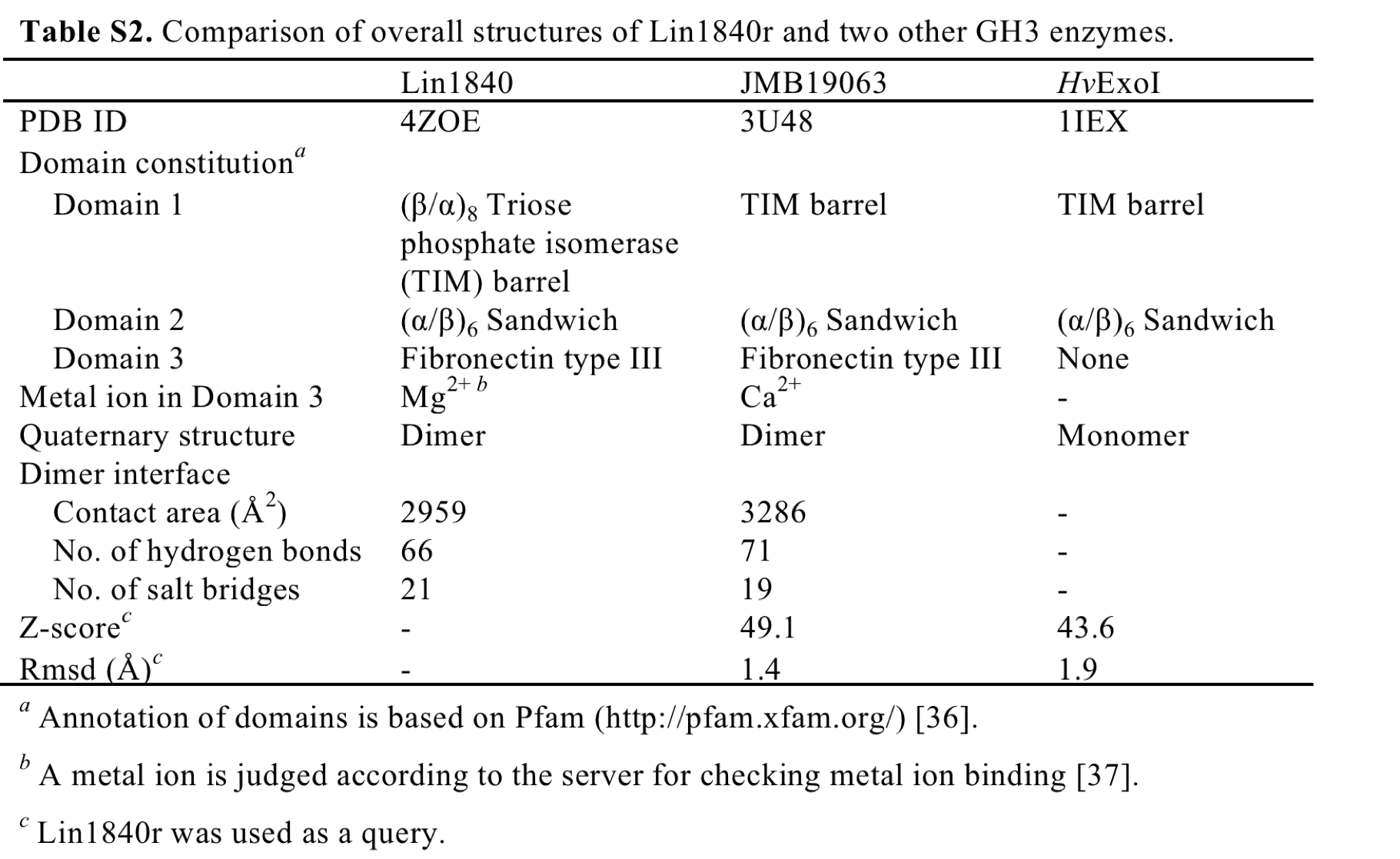

Supplement: S2 Table — (TIF) [file pone.0148870.s006.tif]

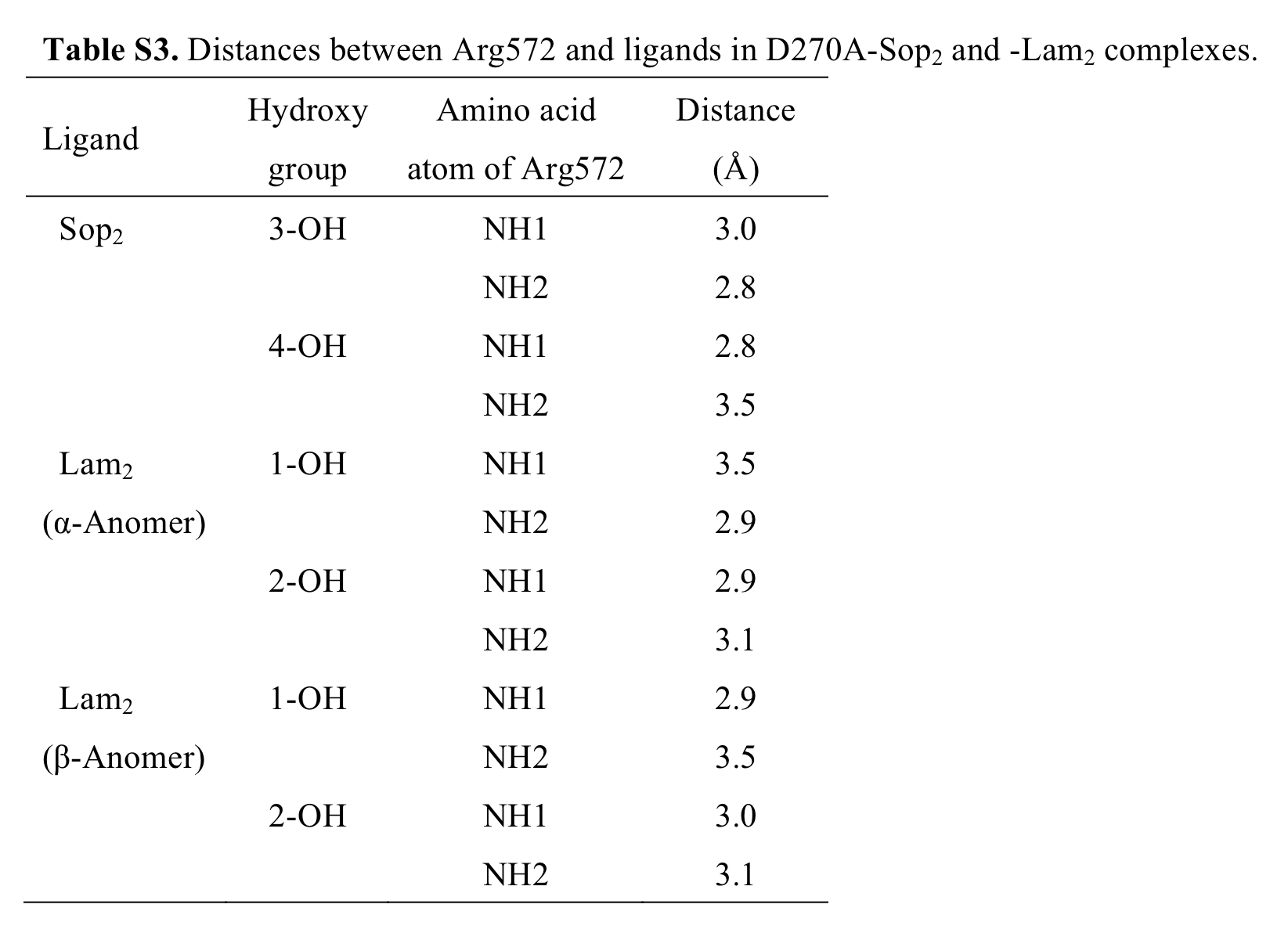

Supplement: S3 Table — (TIF) [file pone.0148870.s007.tif]
